# Removal of Indoor Volatile Organic Compounds via Photocatalytic Oxidation: A Short Review and Prospect

**DOI:** 10.3390/molecules21010056

**Published:** 2016-01-04

**Authors:** Yu Huang, Steven Sai Hang Ho, Yanfeng Lu, Ruiyuan Niu, Lifeng Xu, Junji Cao, Shuncheng Lee

**Affiliations:** 1 Key Lab of Aerosol Chemistry & Physics, Institute of Earth Environment, Chinese Academy of Sciences, Xi’an 710061, China; stevenho@hkpsrl.org (S.S.H.H.); luyanfeng86@126.com (Y.L.); nrynrj@126.com (R.N.); xulif6789@163.com (L.X.); 2 State Key Lab of Loess and Quaternary Geology (SKLLQG), Institute of Earth Environment, Chinese Academy of Sciences, Xi’an 710061, China; 3 Division of Atmospheric Sciences, Desert Research Institute, Reno, NV 89512, USA; 4 Department of Civil and Environmental Engineering, The Hong Kong Polytechnic University, Hung Hom, Hong Kong, China; shun-cheng.lee@polyu.edu.hk

**Keywords:** VOCs, formaldehyde, photocatalysis, review, influencing factors

## Abstract

Volatile organic compounds (VOCs) are ubiquitous in indoor environments. Inhalation of VOCs can cause irritation, difficulty breathing, and nausea, and damage the central nervous system as well as other organs. Formaldehyde is a particularly important VOC as it is even a carcinogen. Removal of VOCs is thus critical to control indoor air quality (IAQ). Photocatalytic oxidation has demonstrated feasibility to remove toxic VOCs and formaldehyde from indoor environments. The technique is highly-chemical stable, inexpensive, non-toxic, and capable of removing a wide variety of organics under light irradiation. In this paper, we review and summarize the traditional air cleaning methods and current photocatalytic oxidation approaches in both of VOCs and formaldehyde degradation in indoor environments. Influencing factors such as temperature, relative humidity, deactivation and reactivations of the photocatalyst are discussed. Aspects of the application of the photocatalytic technique to improve the IAQ are suggested.

## 1. Introduction

As more illnesses are being attributed by indoor air pollution, indoor air quality (IAQ) of residential units and workplaces is a serious concern. Human beings spend >80% of lifetime indoors, including living and working places such as dwellings, offices, and workshops [1,2]. Typical indoor air pollutants are particulate matters (PM), nitrogen oxides (NO_x_), carbon monoxide (CO), and volatile organic compounds (VOCs). Among those, VOCs [3,4] is one class of prominent and representative indoor pollutants. The United States Environmental Protection Agency (U.S. EPA) estimated that the VOCs levels in indoor air are typically 5–10 times higher than those of outdoor air [5]. Currently, over 50% of the precedent-controlled pollutants proposed by the U.S.EPA are VOCs [6]. One such dangerous VOC that is being targeted by the Toxic Substances Control Act (TSCA) is formaldehyde. Formaldehyde is particularly important because of its ubiquitous presence and various adverse effects on human health. Furthermore, it is a challenge to collect and quantify formaldehyde in the air due to its higher polarity and reactivity compared with other VOCs. Distinct monitoring and measurement methods are thus required. The most commonly used offline method for simultaneous determination of formaldehyde is to collect the carbonyls on solid sorbents coated with a suitable derivatization agent (e.g., 2,4-dinitrophenylhydrazine (DNPH)), followed by solvent desorption and liquid injection for analytical analysis (e.g., by high-pressure liquid chromatography (HPLC)) [7,8].

Many VOCs are ubiquitous in the indoor environment in view of the presence of typical indoor emission sources [2,9,10]. Indoor VOCs are produced from a variety of sources, including the utilization of consumer household products, emissions from adhesives and building materials, and combustion processes [10,11,12]. VOCs are easily absorbed by the skin and mucous membranes, causing damaging consequences to human organs and metabolic systems. A few VOCs are also linked with sick building syndrome (SBS) [13,14]. Formaldehyde is one of the representative oxygenated-VOCs. More than 65% of global formaldehyde is used to synthesize resins such as urea-formaldehyde (UF), phenol-formaldehyde (PF), and melamine-formaldehyde (MF), which are widely used in the construction materials, wood processing, furniture, textiles, carpeting, and chemical industries [15]. In addition, it is strongly persistent and thus can slowly release from the materials over an extensive period [4]. Formaldehyde is classified as a human carcinogen and it has been given more attention because of its adverse health effectss [16]. Therefore, the removal of indoor VOCs and formaldehyde in particular is of widespread interest in view of avoiding the potential imposed adverse effects on human health.

Emission source control, ventilation, and air cleaning are the three important approaches to improve indoor air quality [17]. Among these air pollution control strategies, air cleaning with Advanced Oxidation Processes (AOPs) has been drawing more and more attention because of the restraints in the production of secondary pollution. Photocatalysis, as a promising technology developed since 1972 [18], is defined as the process by which various environmental pollutants are degraded on the surface of a semiconductor photocatalyst when exposed to a sufficiently energetic irradiation source, and is an important group of AOPs [19]. The merit of photocatalysis is that it can be operated at room temperature and is capable of degrading many organics under light irradiation. In the past two decades, a lot of studies have been conducted for the photocatalytic oxidation of VOCs and formaldehyde which are beneficial to solve the indoor pollution issues [20]. TiO_2_ has been the dominant photocatalyst because of its superior photocatalytic oxidation ability, high photocorrosion resistance, and non-toxic properties [21]. TiO_2_ immobilized on different substrates can photocatalytically degrade indoor air pollutants in a flow system under UV light irradiation [20,22]. However, TiO_2_ can only be activated by ultraviolet (UV) light because of its large band gap (3.2 eV), and UV light accounts for only 5% of the solar energy [23]. Although dye-sensitized and transition metal-doped or nonmetal-doped TiO_2_ can extend its optical absorption to the visible light range, many researchers have focused their efforts on the development of novel non-TiO_2_ catalysts with low band gaps [24,25,26,27,28]. This interest is due to the fact that stable and efficient dyes are usually rare, whereas dopants can serve as recombination centers for the photogenerated electrons and holes [21]. An alternative method is to combine photocatalysis with other processes that enhance the degradation efficiency. For example, Tokumura and coworkers developed the photo-Fenton reaction for the removal of VOCs which can efficiently prevent emission of any by-products [29]. A compact scrubber and AOP process were combined to enhance the VOC oxidation [30]. The combination of AOPs and gas absorption is able to effectively transform chlorine into chloride ions under ambient temperature conditions [31].

A number of reviews about different aspects of the photocatalytic oxidation of VOCs have been published in recent years. For example, Kabir *et al.*, reviewed some representative techniques for controlling indoor VOCs [32]. Peral *et al.*, discussed the basic phenomena like oxygen and water vapor adsorption during gas-solid heterogeneous photocatalysis, and special interest was taken in describing the different photo-reactor configurations [33]. Lim *et al.*, reviewed the development of photocatalytic materials and photoreactors which significantly affect the degradation efficiency of various major air pollutants [19]. Zaleska *et al.*, reviewed the air pollutant removal mechanisms, key influencing factors on the reaction rate as well as photocatalyst preparation and immobilization techniques [34]. The review by Mo *et al.*, concentrates on the preparation and coating of various photocatalytic catalysts, different kinetic experiments and models, novel methods for measuring kinetic parameters, reaction pathways, intermediates generated, and an overview of various photocatalytic reactors and their models described in the literature [20]. Wang *et al.*, reviewed the current exposure level of VOCs in various indoor environment and state of art technology for photocatalytic oxidation of VOCs from indoor air [35]. Zhong and Haghighat carried out a critical review with aims to examine the state-of-the-art of photocatalysis technologies in the field of air purification and their application prospects [36]. Most recently, Hay *et al.*, reviewed the viability of photocatalysis for air purification, especially the catalyst lifetime and intermediate formation [37].

In this review, we aim to summarize and review the current progress of photocatalytic removal of VOCs in indoor environments. Firstly, emission sources of indoor VOCs and the traditional indoor air pollution control strategies are discussed. Secondly, influencing factors such as temperature, relative humidity, deactivation and reactivations of the photocatalyst are discussed and special interest is paid for the production of intermediates. Further applications of the photocatalytic technique to improve the indoor air quality are suggested.

## 2. VOCs in Indoor Environment

### 2.1. Sources of VOCs Indoors

VOCs is defined as organic compounds with a boiling point in the range of 50–260 °C at room temperature and atmospheric pressure [38]. This group is composed by a large amount of low molecular weight (MW) pollutants (such as aromatic-, fatty-, halogenated-, and oxygenated-hydrocarbon, terpenes, aldehydes, ketones, and esters). Table 1 lists the typical VOCs presented in indoor air and their potential sources [17]. Formaldehyde is colorless, flammable and highly reactive at room temperature.

The concentrations of common VOCs in a given indoor environment strongly related to the existences of emission sources and the efficiency of ventilation. In some cases, indoor VOCs levels are extremely high owing to low air exchange rates (AER) and poor ventilation [39]. For formaldehyde, the atmospheric background mixing ratio is generally at the ppbv to sub-ppbv level, which is much lower than that indoors (e.g., ppmv level) such as workspaces and residential units [40]. VOCs can be generated from indoor sources and can also penetrate from outdoors via air exchange.

**Table 1:** Potential sources of indoor VOCs.

VOCs	Possible Sources
Formaldehyde	Pesticides, flooring materials, insulating materials, wood-based materials, machine, coatings and paints
Toluene	Pesticides, flooring materials, insulating materials, wood-based materials, paints, adhesives, gasoline, combustion sources
Acetaldehyde	Wood-based materials, flooring materials, HVAC system
Paradichlorobenzene	Ceiling materials, wood-based materials, pesticides
Ethylbenzene	Furniture, paints, adhesives, gasoline, combustion sources
Methylene chloride	Flooring materials, furniture, HVAC system, coatings and paints
Chloroethylene	Flooring materials, coatings and paints, dry-cleaned clothes
Carbon tetrachloride	Coatings and paints, industrial strength cleaners
Chloroform	Pesticide, glue
Naphthalene	Insulating materials, mixed materials, wall painting
Other VOCs (e.g., esters and ketones)	Plastics, resins, plasticizers, solvents usage, flavors, perfumes, paints, disinfectants, adhesives

#### 2.1.1. Indoor Sources

Building and decoration materials are the direct emission sources for many common VOCs. In addition, the additives in solvent paints, wood preservatives, and plywood can release different amounts of VOCs at room temperature. Flooring can emit volatile aromatic compounds such as toluene, benzene, and xylene [41]. Acetaldehyde, used as a preservative and food seasoning for fish products, can be released from aniline, cosmetics, and plastic products as well. Newspapers, magazines, and prints that people are regularly expose to are the source of C_8_ aromatics [42]. Furthermore, dry-cleaned clothes, chlorinated water, industrial-strength cleaners and room deodorants are the main source of chlorinated hydrocarbons. Environmental tobacco smoke is an important source for indoor VOCs in which a total of 78 low MW chemical species has been quantified, including aromatics, polycyclic aromatic hydrocarbons (PAHs), carbonyls, and quinones in the cigarette gases [43]. Human metabolism is also a source of indoor VOCs. Acetone, acetaldehyde, methanol and other aldehydes were detectable in the respiratory air [44].

Formaldehyde is a good solvent with strong adhesive properties, thus is used to strengthen plate hardness. In addition, its insect-resistance and anticorrosive ability allow it to be applied in production of urea formaldehyde (UF) resins, paints and other materials. Primary non-industrial indoor sources of formaldehyde include decorative building materials and furniture bonded with UF resins, UF acid-cured finishes, and UF foam insulation (UFFI) such as wood-based materials, flooring and coatings [12,45]. The interior components of furniture and building materials (e.g., floor glue, plywood, emulsion paint, synthetic fiber, and adhesives) can emit a large quantity of formaldehyde. The emission from UF-bonded materials has universality, potentiality and durability [46]. The volatiles are mostly located deep in the plank rather than on the surface, resulting in slow, continuous, and uninterrupted physical release. However, such potential would decrease over time. 

Heat treatment and combustion are also important sources of indoor formaldehyde. Traditional fuels such as biomass, coal, kerosene and liquid petroleum are used as energy sources for in-house warming, especially in most developing countries [47,48]. The heating will no doubt emit a certain amount of formaldehyde and other air pollutants that elevate the toxic levels and create a polluted indoor environment. Residential cooking is considered as an anthropogenic source of indoor formaldehyde [49,50,51]. Daily necessities and customer products such as cosmetics, cleaning detergents, pesticides, chemical fiber textiles, books, and printing ink can release airborne formaldehyde.

#### 2.1.2. Outdoor Sources

Outdoor VOCs can be originated from anthropogenic or natural sources [52,53,54,55,56]. Incomplete combustion processes can generate volatile dissipative of any substances with low boiling point. Automobile exhaust, industrial discharges, and fuel combustion products contain many VOCs represented by alkanes, olefins, aromatic hydrocarbons. The pollutants from oil-fueled automotive include trace amount of rubber matrix, which consist of high numbers of alkanes and alkyl benzene. For the natural sources, biological VOCs (BVOCs) can be formed from secondary metabolic reactions of vegetation [57,58].

Formaldehyde is an intermediate of atmospheric photochemical oxidation and emission product from fossil fuel combustion. The primary sources of formaldehyde include both anthropogenic and natural sources as well. Natural formaldehyde can release from solid wood, forest fire and excretion of animals; however, their contributions to the atmospheric level are relatively small [59]. Anthropogenic emissions include motor vehicles, chemical plants, industries, coal processing, artificial biomass combustion, and food barbecue. Among those, vehicle exhaust (VE) is the most critical pollution in urban areas. Even though alternative fuels and additives (*i.e.*, green energies) and more advanced emission control technology have been discovered to reduce pollutant generation, the raise in amount of oxygenated VOCs from VE is still found with the increasing number of vehicles [60,61]. Formaldehyde can be formed secondarily from oxidation of many VOCs. Alkanes, alkenes and aromatics (e.g., benzene and toluene) are precursors for the photochemical processes [59] which react with atmospheric ozone (O_3_), NO_x_, hydroxyl radical (•OH) resulting in the formation of photochemical smog and production of formaldehyde or other reactive compounds.

**Table 2:** Summary on current control techniques for VOCs removal.

Techniques	Principle	By-Product	Advantage	Disadvantage	Ref.
Botanical purification	Air is passed through a planted soil or directly on the plants. The contaminants are then degraded by microorganisms and/or plants, the precise mechanisms being unclear.	CO_2_, organic and amino acids	Low cost, no secondary pollution, beautifying the indoor environment	The purification effect is bad for high concentration pollutants	[28,62]
Catalytic combustion	Combustion of VOCs at low temperature with the help of a catalyst.	CO_2_, H_2_O	Wide range of application coverage, high efficiency, no secondary pollution	Not suitable for gas containing dust particles and droplets	[63,64]
Bio-filtration	Bio-filtration is a process in which contaminated airs passed through a biological stuffing medium that supports many kinds microorganism that biodegrade the VOCs.	Biomass	Little or no energy needs to be added in the form of heat or radiation to support this process	The equipment is big, long residence time, easy to jam	[65,66]
Absorption	Absorption is used to remove VOCs from gas streams by contacting the contaminated air with a liquid solvent.	Wastewater	Product recovery can offset annual operating costs	High demands on absorbent, complex process, high cost	[24]
Zeolite based adsorption	Air pollutants are adsorbed onto zeolites, often as filtration post-treatment.	Spent zeolite and collected organics	Effective in more than 90% RH as the adsorbent might be too specific	Pollutant reemission	[67]
Activated carbon based adsorption	VOCs are removed from the inlet air by physical adsorption onto the surface of the carbon.	Spent carbon and collected organics	Recovery of compounds, which may offset annual operating costs	They are flammable, difficult to regenerate for high boiling solvents, promote polymerization or oxidation of some solvents to toxic or insoluble compounds, and require humidity control.	[68]
Membrane Separation	Pollutants are passed through a membrane into another fluid by affinity separation.	Exhausted membrane	No further treatment, simple process, small energy consumption, no secondary pollution	The stability of the membrane was poor	[69]

## 3. Traditional Removal Approaches

The traditional technologies for VOCs removal include adsorption, membrane separation, liquid absorption, and catalytic combustion [70]. Many of these techniques have been widely applied in industries or commercial sectors, but few are being further developed or optimized [24,63,67,69]. Table 2 summarizes details of current control techniques for VOCs removal. Newly-developed technologies have demonstrated their removal efficiencies in particular testing airs or controlled environmental chambers. However, many are still limited to theoretical research without practical applications. In addition, single-based removal system may not offer satisfactory purification results due to the complexity of VOCs and variations on their characteristics in real world. Combinations of the technologies are thus required to achieve the final goal, but both high costs and harsh conditions are limitations for their practical applications. There is a need to develop more economic, effective and environmental-friendly treatment methods.

Adsorption is the most traditional method for removal of VOCs. Activated carbon, molecular sieve and silica gel are porous materials with a large surface area medium for physical and chemical adsorption. The common absorbents contain inorganic salts (e.g., ammonium and sulfurous) and are composed with amine groups such as urea and its derivatives, hydrazine, and amino-containing polymers [71,72,73]. Physical adsorption involves VOCs being trapped onto the materials such as zeolites, activated carbon, activated alumina and molecular sieves and porous clay ore without changing their original form. Chemical absorption works with high water solubility VOCs such as formaldehyde, which is then reduced or decomposed by any oxidizing or completing agents in the collection solutions [16]. Persistence and stability are two concerns for the absorber of aldehyde material (ACM; [74]). The absorbed gases should be re-released subject to any change of indoor conditions such as temperature and RH.

Catalytic oxidation technology with thermal treatment is another effective method for VOCs removal. Formaldehyde reacts with oxygen (O_2_) over noble metals producing CO_2_ and H_2_O vapor [40]. The energy consumption cost is a critical concern as this has to be operated at high operation temperatures. For the plasma catalytic method, the molecules, particles, atoms and free radicals are excited to have high chemical activities for the decomposition of VOCs, but the reactions are difficult to control in normal conditions and the reaction rates are usually slow [75]. The feasibility of microbial degradation for removal of formaldehyde in both wastewater and exhaust gas from industries and laboratories has been demonstrated. Currently this technique is not widely applied for the indoor air cleaning. A composite of biological enzyme(s)/activated carbon fiber was synthesized and loaded on an AC surface [76]. Acidity is the most important factor in selecting proper biological enzymes for the degradation. The experimental results showed that the removal rate of formaldehyde reached 80% when the loading time was 8 h.

## 4. Removal of Indoor VOCs and Formaldehyde via Photocatalytic Oxidation

### 4.1. Removal of VOCs by Photocatalytic Oxidation

Photocatalytic oxidation (PCO) has attracted more attention because of its unique characteristics on the removal of chemicals. In recent years, PCO has been perceived as a technology to remove indoor VOCs. Titanium dioxide (TiO_2_) is known as the most extensive studied photocatalyst due to its excellent stability, high photo-activity, and suitable band gap structure. Low cost and non-toxicity are also the main advantages for its application.

The basic mechanism of photocatalytic degradation is that organics would be oxidized to H_2_O, CO_2_ or any inorganic harmless substances with •OH or superoxide (•O_2_^−^) radicals, which are generated on the surface of photocatalyst (e.g., TiO_2_) under ultra-violet (UV) light irradiation [77]:

(1) $${\mathrm{TiO}}_{2}+h\upsilon \to h_{\mathrm{VB}}+e_{\mathrm{CB}}^{-}$$

(2) $${\mathrm{TiO}}_{2}+h_{\mathrm{VB}}^{+}\to •\mathrm{OH}+H^{+}$$

(3) $$O_{2}+e_{\mathrm{CB}}^{-}\to •O_{2}^{-}$$

In the heterogeneous reaction system, TiO_2_ is excited by the absorption of a photon with energy greater than or equivalent to the band gap energy of the semiconductor, resulting in the electron transition from the valence band to the conduction band. The radiation could consequently produce electrons and holes (e^−^/h^+^) in conduction band and valence band, respectively. Following the irradiation, the electrons and holes can undergo redox reactions with the adsorbed reactants on the photocatalyst’s surface that lead to the formation of intermediates and products. The reaction series are the so-called complete mineralization. Besides VOCs degradation, the reactions can be used as a method of disinfection and sterilization [78,79].

**Scheme 1:**
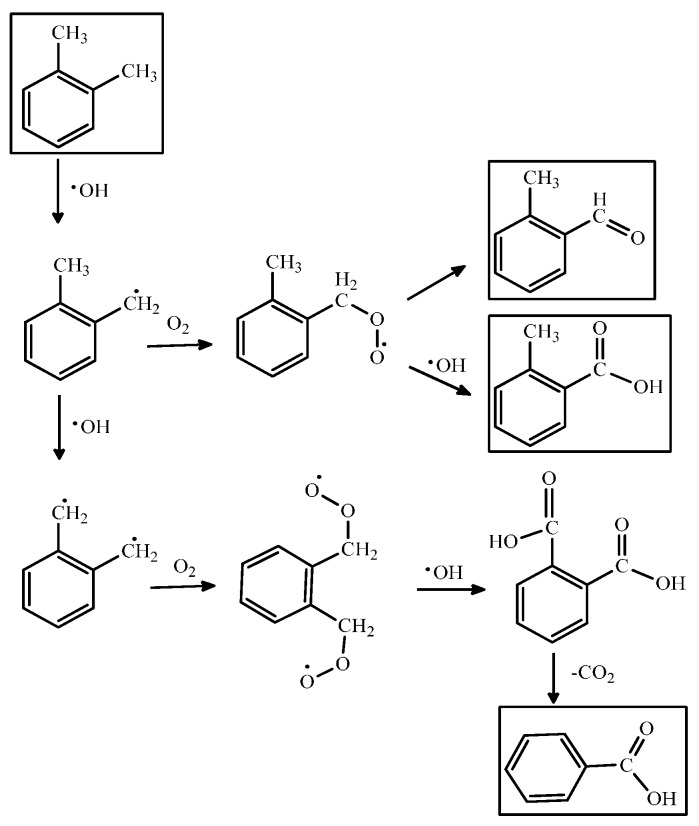
The PCO reaction mechanism for *o*-xylene.

PCO of VOCs consists of a chain of stepwise reactions; that is, they take more than one elementary step to complete. Scheme 1 shows a series of PCO reaction mechanisms for *o*-xylene. Besides the final oxidized products, the steps also yield different oxidation states of the intermediates such as aldehydes, ketones or organic acids [80]. These compounds can be qualified by real-time or offline monitoring and analytical methods such as gas chromatography/flame ionization detection (GC/FID), GC/mass spectrometry (GC/MS), high pressure liquid chromatography (HPLC), and Fourier-transform infrared spectroscopy (FTIR) [81,82]. Table 3 lists the intermediates formed in the PCO of VOCs (e.g., benzene, toluene and xylene) shown in the literature.

**Table 3:** Summary on the intermediates formed in photocatalytic oxidation of typical indoor VOCs.

Target VOC	Concentration (ppm)	Light Source	Main Intermediates	Chemical	Analytical Method	Ref.
Benzene	3000–6000	4000 W Xe lamp	Benzaldehyde, benzoic acid	-	GC/MS	[83]
	614	White fluorescent lamp	Phenol	Hydroquinone, 1,4-benzoquinone	GC/MS	[84]
	-	-	Phenol, hydro-quinone, benzoic acid	Malonic acid, benzoquinone	GC/MS/FTIR	[85]
Toluene	10	Black light lamp	Benzaldehyde, benzoic acid	Benzyl alcohol	FTIR	[86]
	50–800	365 nm UV	Acetone, acetaldehyde, formaldehyde	Acrolein, butanone	TDS-GC/MS/FID, HPLC/UV/FTIR	[87]
	370	>400 nm	Benzaldehyde, benzoic acid	-	DRIFTS	[88]
Xylene	3000–6000	4000 W Xe lamp	Benzaldehyde, Methyl-benzaldehydes	2,5-Furandione, 1,3-isobenzofurandione	GC/MS	[83]
	25–75	UV	*o*-Tolualdehyde, *o*-toluic acid, benzoate ion	-	FTIR	[89]

For instance, the highly stable aromatic ring of toluene usually remains intact while its active methyl group can be oxidized step-by-step to benzoic acid. The formation of the carbonyl group even makes the phenyl ring more inert because the conjugation effect reduces its electron density. The complete oxidation products such as CO_2_ and H_2_O would be generated from any of the intermediates until the phenyl ring is broken. However, if PCO are conducted at room temperature, the active sites on the photocatalyst’s surface could be gradually occupied by irreversibly chemisorbed intermediates, which retard the reactions. For example, during the photocatalytic oxidation processes for toluene over TiO_2_ catalysts, it was found that the toluene photooxidation behavior was strongly affected by the formation and oxidation behavior of intermediate compounds [90]. The study carried out by Nakajima *et al.*, showed that H_2_SO_4_ treatment of TiO_2_ surface provides higher photocatalytic removal efficiency on toluene which can be ascribed to the fast decomposition of intermediates by surface strong acid itself [91]. Moreover, the progresses of the research carried out into TiO_2_-based photocatalysts were summarized by several recent reviews [21,92].

Anatase and rutile, two crystalline phases of TiO_2_, have been shown their feasibilities for PCO of indoor air pollutants under UV light irradiation. The band-gap energies of anatase and rutile are 3.23 and 3.02 eV, respectively. Anatase has shown better performance in PCO processes than that of rutile because of its more favorable conduction band configuration and stable surface peroxide groups. In general, TiO_2_ is fixed on some substrate, such as hollow tubes, silica gel, beads, and woven fabric. These catalysts can be obtained using the methods such as electrochemical [93], plasma deposited [94], dip coating and sol-gel method [95].

Table 4 summarizes potential photocatalysts used for removal of indoor VOCs. Different single or combined photocatalysts have particular removal rates and efficiencies in PCO. Most TiO_2_-based catalysts have optimized performance on near-UV light region because of its large energy band gap between electron-hole pairs of ~3.2 eV. A light source at a wavelength (λ) of <387 nm is required to overcome the gap, understanding that the PCO can only uptake *ca.* 3% of the sunlight [96]. Therefore, a limited number of TiO_2_ catalysts can exhibit high degradation activity under a visible light. A lot of works have been thus done on the improvement of TiO_2_ photocatalytic efficiency, such as doping with nonmetals and metals and coupling with other supports. TiO_2_ doping with a nonmetal atom can enhance the photo-response in a practical application [97]. The nonmetal can substitute the oxygen on TiO_2_ lattice and lead to a band gap narrowing, resulting in activation at far-visible light region. The common photocatalysts are primarily metal oxides, which can be doped with elements such as carbon (C), nitrogen (N) or transition metal ions. For instance, the nitrogen-doped catalysts can be activated more efficiently because of higher energy level of the valence band of N2p than O2p. The fluorescence-assisted TiO_2−x_N_y_ can decompose pollutants such as acetaldehyde through gaseous phase photocatalytic reaction [98]. CaAl_2_O_4_: (Eu, Nd)/TiO_2−x_N_y_ composite is able to store and release energy to continuously maintain the visible-light response to TiO_2−x_N_y_, even in the darkness. Such a property allows the fluorescence-assisted photocatalysts to function at night without supplying extra light sources.

**Table 4:** Summary on potential photocatalysts applied for indoor VOCs removal.

Photocatalyst	Preparation/Coating Method	Configuration	Compounds	Light Source	η_removal_ (%)	Ref.
TiO_2_	Sol-gel	F	Acetone, toluene *p*-xylene	UV lamp, 254 nm	77–62 (3 L/min)	[95]
TiO_2_	Electrochemical	F	Acetaldehyde	UV	99+ (110 min)	[93]
TiO_2_	Sol-gel	F	Toluene	Black light	52 (3.6 L/min)	[86]
TiO_2_	Plasma deposited	F	*m-*Xylene	UV lamp	99+ (30 min)	[94]
TiO_2−x_N_x_	Calcination	P	Toluene	Visible light	99+ (3000 min)	[82]
TiO_2−x_N_x_	Hydrothermal	P	Acetaldehyde	Fluorescence	-	[98]
C-TiO_2_	Hydrothermal	P	Toluene	Visible light	60+ (120 min)	[106]
C-TiO_2_	Hydrothermal	P	Toluene	Visible light	20 (120 min)	[107]
CNT-TiO_2_	Hydrothermal	P	Styrene	UV-LED, 365 nm	50 (20 mL/min)	[108]
Pt/TiO_2_	Photo-deposition	P	Benzene	Black light, 300–420 nm	100 (100 mL/min)	[99]
Ln^3+^-TiO_2_	Sol-gel	P	Benzene, toluene, ethylbenzene, *o*-xylene	UV, 365 nm	22–79	[109]
Ce-TiO_2_	Sol-gel	F	Toluene	Visible light	90	[110]
Fe-TiO_2_	Sol-gel	P	*p*-Xylene	Visible light—LED	22 (5 min)	[111]
Fe-TiO_2_	Sol-gel	P	Toluene	Visible light	99+ (120 min)	[88]
In(OH)_3_	Ultrasound radiation	P	Acetone, Benzene, Toluene	UV lamp, 254 nm	99+ (5 h)	[104]
β-Ga_2_O_3_	Chemical deposition	P	Benzene	UV-lamp, 254 nm	60 (20 mL/min)	[105]
Ag_4_V_2_O_7_/Ag_3_VO_4_	Hydrothermal	P	Benzene	White fluorescent lamp	99+ (120 min)	[84]
Pt/WO_3_	Photo-deposition	P	DCA, 4-CP, TMA	Visible light, >420 nm	99+ (3 h)	[112]
Pd/WO_3_	Calcination	P	Acetaldehyde, toluene	Fluorescence/visible light	99+ (3 h)	[26]

DCA: dichloroacetate; 4-CP: 4-chlorophenol; TMA: tetramethylammonium; P: powder; F: film.

The TiO_2_-Pt/TiO_2_ hybrid catalyst system allows a complete oxidation of benzene to CO_2_ at ambient temperature [99]. TiO_2_ after doping with Pt has an increased number of active sites, which convert the intermediate form of carbon monoxide (CO) into CO_2_. Pt/TiO_2_ is thus the most useful catalyst for the purification of VE gases containing benzene. Doping with lanthanide ions can promote the formation of oxygen vacancies which have relatively high solubility compared with other oxygen species [100]. In particular, cerium (Ce) is a low cost photocatalyst that has the ability to migrate between Ce^4+^ and Ce^3+^ through oxidization and reduction reactions. Ce doped with TiO_2_ can decompose toluene under a visible light source.

Aside from TiO_2_-based photocatalysts, other semiconductors can be also applied in the removal of VOCs such as ZnO [101], ZnS [102], SnO_2_ [103], In(OH)_3_ [104], and β-Ga_2_O_3_ [105]. Nano-sized porous In(OH)_3_ and porous Ga_2_O_3_ have high activity and long-term durability for photocatalytic decomposition of acetone, benzene, toluene and other aromatic derivatives under ambient conditions.

### 4.2. Removal of Formaldehyde by Photocatalytic Oxidation

Similar to the PCO for VOCs, formaldehyde priorly reacts with •OH, which are generated on the excited photocatalyst’s surface. They would form an intermediate of HCOOH which eventually is oxidized to CO_2_ and H_2_O vapor. The reaction mechanism is as follows [113]:

(4) $$\mathrm{HCHO}+•\mathrm{OH}\to •{\mathrm{CHO}+H}_{2}O$$

(5) •CHO+•OH→HCOOH

(6) $$•\mathrm{CHO}+•O_{2}^{-}\to {\mathrm{HCO}}_{3}^{-}\overset{+H^{+}}{\to }\mathrm{HCOOH}\overset{+\mathrm{HCOOH}}{\to }\mathrm{HCOOH}$$

(7) $$\mathrm{HCOOH}\overset{{-H}^{+}}{\to }{\mathrm{HCOO}}^{-}\overset{h^{+}}{\to }H^{+}+•{\mathrm{CO}}_{2}^{-}$$

(8) $$•{\mathrm{CO}}_{2}^{-}\overset{\begin{array}{ccc} \left[O\right] & \left[•\mathrm{OH}\right] & \left[h^{+}\right] \end{array}}{\to }{\mathrm{CO}}_{2}$$

TiO_2_ and TiO_2_-based (*i.e.*, metal-doped, nonmetal-doped and composites), other metal oxides (e.g., MnO_x_, Bi_2_O_3_, ZnO, PdO, and composites), and new-type photocatalysts are widely used for PCO of formaldehyde. Table 5 shows a summary of the common photocatalysts and their applications and efficiency in the formaldehyde decomposition.

**Table 5:** Summary of the PCOs used for formaldehyde degradation.

Catalyst	Preparation Method	HCHO Concentration	Light Source	Conversion Efficiency	Ref.
Mesoporous TiO_2_	Evaporation-induced self-assembly	30 ppm	UV light	95.8%	[114]
Amorphous TiO_2_ film	CVD method	50–55 ppm	UV light	80%	[115]
PEG modified TiO_2_ film	Sol-gel method	20 ppm	UV light	95%	[116]
TiO_2_ coating on polyester fiber	Spray coating	24.6 ± 2.8 ppm	UV light	90%	[117]
UV/TiO_2_/O_3_	Sol-gel	18 ppm	UV light	79.4%	[118]
Ag/TiO_2_	Incipient wet impregnation	500 ppm	UV light	Above 95%	[119]
Pt@TiO_2_	Reverse micelle sol-gel	10 ppm	Vis light	98.3%	[120]
Ce/TiO_2_	Sol-gel	1 ppb	UV light	Above 70%	[121]
Pd-TiO_2_ film	Sol-gel dip coating	500 ppb	UV light	Above 95%	[122]
Acrylic-silicon/nano-TiO_2_	Emulsion blend	0.8 ppm	Vis light	83.4%	[123]
N-doped TiO_2_ film	Precipitation-peptization	0.24 ppm	Vis light	90%	[124]
AC loading TiO_2_	Microwave-assisted synthetic	30 ppm	UV light	58.68%	[22]
Pt@SnO_2_	Sol-gel method	—	Vis light	93.2%	[125]
α-Bi_2_O_3_	Calcination of hydrothermally prepared (BiO)_2_CO_3_	100 ppm	Vis light	62.5%	[25]
Nano-ZnO	Mixing-calcination	2.5–25 ppb	UV light	73%	[126]
Zr_0.08_Ti_0.92_O_2_	Sol-gel method	0.08 ppb	UV-vis light	92%	[127]
Zn_2_SnO_4_	Hydrothermal method	2 ppm	UV-vis light	70%	[128]

### 4.3. Influencing Factors

Photocatalytic reaction rate, additional with the reaction kinetic and adsorption coefficients, are direct tools to evaluate the efficiency of a photocatalyst in removal of VOCs. Table 6 shows kinetic parameters and PCO conversion efficiency for the common VOCs. There are critical factors such as light source and intensity, pollutant concentration, RH, temperature, and deactivation and reactivation which can control the photocatalytic reaction rate. In order to study the PCO processes, many kinetic experiments for removal of common pollutants (e.g., benzene, toluene, xylene, and formaldehyde) have been thus conducted in optimal reactors. Here we summarize and review these factors.

*Light source and intensity*. The electron-hole pairs of a photocatalyst must be firstly excited for the subsequent VOCs degradation. The common catalysts (e.g., TiO_2_) usually require an UV wavelength equivalent energy source for the excitation. Medium pressure mercury lamps, xenon lamps, and UV lights are common light sources for PCO. The light intensity is usually represented by units of light-irradiation (energy per unit area) or photon flux on the catalyst’s surface. Theoretically, the reaction rate of PCO is proportional to the intensity of the light supply. The reaction rate of PCO is regulated by the first order of consumption rate of electron hole pairs and a half order of their recombination rate [129]. Thus there is no doubt that the light intensity can directly control the first-order of reaction [95]. In addition, the internal structures of photocatalysts can affect the adsorption rate of the photons and consequently impact the conversion rate [130]. Bahnemann and Okamoto [131] investigated the relationships between UV light intensity and photocatalytic reaction rate with TiO_2_. A linear correlation was found in the low intensity range whereas the degradation rate is proportional to square root of the light intensity under the moderate intensities. When light intensity is greater than 6 × 10^−5^ Einstein L^−1^·S^−1^, the VOC degradation rate is not further enhanced subject to any changes.

As UV light is harmful to human and potentially leads to produce secondary pollutants (e.g., more strong oxidizing substances) in indoor air, more attention is being paid to applying visible light stimulating catalytic reaction for the removal of VOCs. However, the influences of light intensity are seldom studied with visible light sources. The formaldehyde removal rates with N-doped TiO_2_ photocatalyst were enhanced linearly form 25.5% to 59.6%, and stabilized thereafter, when the intensity increased to 30,000 lux with an initial concentration of 0.98 mg/m^3^ [132].

**Table 6:** Kinetic parameters and PCO conversion efficiency (%) for the common VOCs.

Pollutants	Reactor Design		Initial Reaction Conditions				Deactivation	Ref.
	RT	Photocatalyst	[VOC] Gas (ppm)	PW(nm)/I (mW·cm^−2^)	RH (%)	T (°C)		
Styrene	CR	CNT-TiO_2_	25 ± 1.5	365/70	-	-	Y	[108]
Benzene	CR	Pt/TiO_2_	80	300–420/-	65	Ambient	n.r.	[99]
	CR	In(OH)_3_	920	245/-	-	25	n.r.	[104]
Acetone	CR	In(OH)_3_	420	245/-	-	30 ± 1	n.r.	[104]
Toluene	CR	TiO_2_	10	>300/0.7	0–40	Ambient	Y	[86]
	CR	TiO_2_	17–35	365/2.34	47	25	n.r.	[104]
	CR	P25	50–800	365/10 ± 1	0–50	25	n.r.	[87,104]
	CR	Ce-TiO_2_	0.15–0.6	Visible/-	<3–75	42	n.r.	[110]
	CR	Fe-TiO_2_	370	>400/-	60	25	Y + N	[88]
	CR	Ln^3+^-TiO_2_	23 ± 2	365/0.75	-	-	n.r.	[109]
	CR	In(OH)_3_	1220	245/-	-	25	n.r.	[104]
	CR	TiO_2_ fibers	200	365/9	20–60	-	n.r.	[133]
Xylene	CR	P25	25–75	UV/1.5	30–90	-	Y	[89]

CR: continuous reactor; BR: batch reactor; [VOC] gas = VOC gas-phase concentration; I = light intensity; RH = relative humidity; T = temperature; Y: catalyst deactivation observed; N: catalyst deactivation not observed; Y + N: catalyst partial deactivation and can be regenerated completely; n.r.: reference includes no data on catalyst deactivation; -: reference includes no data on light intensity.

*Pollutant concentration*. The concentration levels of pollutants can influence the photocatalytic performance in terms of the reaction rate. In the PCO process, the mass flux between the surface of photocatalyst and inlet can be accounted by the convective mass transfer [134]:

N_A_ = k_A_ × ∆C_A_ (9)

where N_A_ is mass flux, k_A_ is convective mass transfer coefficient and ∆C_A_ is the concentration difference of transfer substance between the interface and the inlet. Eventually, the pollutant concentration over the photocatalyst’s surface varies from that in the inlet; however, it is difficult to accurately monitor the surface concentration by any means of measurement techniques. As a result, the use of inlet concentration for the computation of kinetic parameters may contain different degrees of errors. In order to decrease the concentration disparities, it is necessary to increase the airflow rate for improving the convective mass transfer [135].

Pollutant concentration (C) and photocatalytic reaction rate (r) are the two kinetic parameters for reaction model computation. The Langmuir-Hinshelwood (L-H) model has been widely applied to establish the relationship between C and r in the PCO process for many VOCs such as acetone, benzene, toluene, and xylene [136,137]. In general, the degradation rate decreases when the pollutant concentration increases [87,95]. However, only a few investigations on the photocatalytic kinetics for indoor VOCs are reported. Among those, most have conducted the tests at an extremely high concentration (e.g., ppmv level). This demonstration concentration for a VOC could even cause instant headaches, irritation, and discomfort to humans [138]. The results cannot reflect the realistic situation in most indoor environments (*i.e.*, pptv to sub-ppbv level). Ce-doped TiO_2_ had a decrease in degradation efficiency while the formaldehyde levels increased from 0.1 to 0.5 mg/m^3^ [121]. In addition, in a concentration range of 0.1–1.0 mg/m^3^, the degradation efficiency of formaldehyde was up to 80.8% with a photocatalyst from the 3M Company, but was sharpely reduced to 52.9% when the concentration raised to 2.0 mg/m^3^ [139].

*Relative Humidity*. Hydroxyl groups can be generated from water molecules adsorbed on the photocatalyst during the PCO processes, which can be captured by photo-generated charge carriers to produce reactive radicals (e.g., •OH) to further oxidize the indoor organic pollutants. Therefore, water vapor either from indoor air or generated from the mixed reactions plays a significant role in the photo-degradation process [99]. In the absence of water vapor, the photo-degradation of VOCs (e.g., toluene) is seriously retarded since the mineralization could not completely occur. At the initial stage of the photocatalytic reaction, hydroxyl groups are expended due to the reactions between water vapor and organics on the photocatalysts’ surfaces. However, the presence of water vapor would lead to electron-hole recombination [140]. There is also an adsorption competition between water and organics when RH is excessive. The water molecules can hide the active sites of the photocatalyst surface and so reduce the VOCs degradation rate and the photocatalytic activity. A typical breakthrough curve was obtained to demonstrate the competitive adsorption of water and toluene in the TiO_2_ photocatalytic reactions [86]. The result indicated that the photocatalyst is more sensitive to RH under a low hydrophobic condition. The indoor RH is usually regulated by ventilation (e.g., air-conditioning) or humidifiers, thus the competitive adsorption between water and trace contaminants has a strong impact on the oxidation rates [135].

RH is also the key factor for formaldehyde degradation, which has been demonstrated with the photocatalytic performances of Zr_x_Ti_1−x_O_2_ at different RHs of 50% ± 5%, 65% ± 5%, 85% ± 5%, respectively [127]. The work reported that the activity is the highest at RH of 50% ± 5%, representing that the photocatalytic reaction can be suppressed at humid environments. Similar observations were found for TiO_2_-C coated and TiO_2_-CN coated photocatalysts at a RH range of 20%–90% [141]. The effect of RH on the degradation is negligible at a formaldehyde concentration of 3.3 ± 0.3 ppmv; while at a higher concentration level (8.6 ± 0.5 ppmv), the degradation efficiency significantly dropped at a RH of 90%. It is necessary to note that the impacts of water vapor on the removal efficiency for VOCs and formaldehyde were inconsistent for different photocatalysts. For this reason, an optimized working RH must be investigated when different systems are applied.

*Deactivation and reactivation*. Lifetime of a photocatalyst is an important parameter for the real application in removal of indoor pollutants. This should include the consideration of deactivation, regeneration, reactivation, or replacement. The gas-solid photocatalytic activity decreases with time while the number of effective active sites on the catalyst surface decreases at the same time. Deactivation thus occurs due to the accumulation of such partially-oxidized intermediates which occupy the active sites on the photocatalyst’s surface. Many kinetic studies indicate that the adsorption of poisonous intermediates during the initial stage of the photocatalytic reactions is almost irreversible. The initial oxidation rate is proportional to the effective surface area of catalyst. For instance, acetic acid and formic acid are the two main detectable intermediates formed in the photocatalytic degradation of acetaldehyde by TiO_2_. Even though trace amounts of these intermediates could possibly discharge into the airs, these polar organic compounds have a stronger affinity to be accumulated on the photocatalyst’s surface until they can be decomposed by further steps of PCO. In some extent, a complete deactivation of the photocatalyst occurred after 20 consecutive PCO reactions due to the fully occupation of the active sites by the intermediates [89]. Mendez-Roman investigated the relationship between the formation of surface species and catalyst deactivation during the photocatalytic oxidation of toluene, and their results showed that the accumulation of benzoic acid on the surface resulting in the catalyst deactivation [142]. Recovery of photocatalytic activity requires a regeneration technique. The adsorbed polar intermediates such as benzaldehyde and benzoic acid can be removed completely with a heat treatment at 653 K for 3 h [107]. However, such reactivation of the photocatalysts is a practically difficult since it consumes high energy or requires working with a furnace.

*Other potential factors*. Rather than the above, the loading amount of noble metal, content of the photocatalyst, and gas flow rate can also affect the photocatalytic activity. These multiple parameters can either advance or suppress the PCO subject to the kind of photocatalysts applied for the VOCs removal.

## 5. Summary and Outlook

VOCs are omnipresent and can greatly aggravate indoor air quality. Formaldehyde is of high concern due to its carcinogenicity and universality. There is a variety of indoor VOC pollution sources such as wood-based furniture and flooring materials. A long exposure to indoor toxics can lead to health impacts such as SBS and cancer.

Photocatalysis is considered as one of the most promising technologies for eliminating VOCs due to its high efficiency and stability. However, traditional photocatalytic materials such as TiO_2_ can only respond to UV irradiation, limiting the light utilization efficiency. Development of new single or photocatalytic composite materials which can be irridiated with conventional visible or solar light is thus a need. Currently most studies demonstrate their VOCs removal efficiency in a high concentration level (e.g., ppmv). More on-site demonstrations should be conducted in order to prove the efficiency in removal of indoor VOCs in realistic environments (e.g., residential and work spaces).

Different oxidation states of intermediates can be produced in the PCO reaction mechanism. These organics can temporarily or permanently occupy the active sites on the photocatalyst’s surface, leading to suppression or termination of the reaction kinetics. Efficient removal of these intermediates is necessary as they are often even more toxic than the parent VOCs and harmful to health. It is especially critical if they can be discharged into the indoor airs in practical applications. More advanced approaches for re-activation and regeneration of photocatalysts are also essential to extend their lifetime to allow long-term VOC degradation.
